# Tucidinostat Plus Exemestane as a Neoadjuvant in Early-Stage, Hormone Receptor-Positive, Human Epidermal Growth Factor Receptor 2-Negative Breast Cancer

**DOI:** 10.1093/oncolo/oyae033

**Published:** 2024-03-09

**Authors:** Hongmeng Zhao, Dan Li, Qian Li, Bin Zhang, Chunhua Xiao, Ying Zhao, Jie Ge, Yue Yu, Yumian Jia, Xiaojing Guo, Xuchen Cao, Xin Wang

**Affiliations:** The First Department of Breast Cancer, Tianjin Medical University Cancer Institute and Hospital, National Clinical Research Center for Cancer, Tianjin 300060, People’s Republic of China; The First Department of Breast Cancer, Tianjin Medical University Cancer Institute and Hospital, National Clinical Research Center for Cancer, Tianjin 300060, People’s Republic of China; The First Department of Breast Cancer, Tianjin Medical University Cancer Institute and Hospital, National Clinical Research Center for Cancer, Tianjin 300060, People’s Republic of China; The First Department of Breast Cancer, Tianjin Medical University Cancer Institute and Hospital, National Clinical Research Center for Cancer, Tianjin 300060, People’s Republic of China; The First Department of Breast Cancer, Tianjin Medical University Cancer Institute and Hospital, National Clinical Research Center for Cancer, Tianjin 300060, People’s Republic of China; The First Department of Breast Cancer, Tianjin Medical University Cancer Institute and Hospital, National Clinical Research Center for Cancer, Tianjin 300060, People’s Republic of China; The First Department of Breast Cancer, Tianjin Medical University Cancer Institute and Hospital, National Clinical Research Center for Cancer, Tianjin 300060, People’s Republic of China; The First Department of Breast Cancer, Tianjin Medical University Cancer Institute and Hospital, National Clinical Research Center for Cancer, Tianjin 300060, People’s Republic of China; The Department of Breast Pathology, Tianjin Medical University Cancer Institute and Hospital, National Clinical Research Center for Cancer, Tianjin 300060, People’s Republic of China; The Department of Breast Pathology, Tianjin Medical University Cancer Institute and Hospital, National Clinical Research Center for Cancer, Tianjin 300060, People’s Republic of China; The First Department of Breast Cancer, Tianjin Medical University Cancer Institute and Hospital, National Clinical Research Center for Cancer, Tianjin 300060, People’s Republic of China; The First Department of Breast Cancer, Tianjin Medical University Cancer Institute and Hospital, National Clinical Research Center for Cancer, Tianjin 300060, People’s Republic of China

**Keywords:** tucidinostat, hormone receptor-positive, neoadjuvant treatment, Ki67

## Abstract

**Background:**

To assess the efficacy and safety of tucidinostat plus exemestane as a neoadjuvant strategy in early-stage breast cancer.

**Methods:**

This prospective, open-label, single-arm phase II trial enrolled patients with stage II-III breast cancer with hormone receptor-positive and human epidermal growth factor receptor 2 (HER2)-negative. Eligible patients received tucidinostat plus exemestane, and then breast-conserving surgery (BCS) or modified radical mastectomy.

**Results:**

Among 20 enrolled patients, 3 of them achieved preoperative endocrine prognostic index (PEPI) score of 0. Additionally, complete cell cycle arrest was observed in 7, radiologic objective response rate in 10, and disease control rate in 20 patients, pathological complete response in 1 patient, and 5 patients performed BCS. Ki67 suppression from baseline to surgery was observed in 17 of patients, with the Ki67 change ratio of −73.5%. Treatment-emergent adverse event included neutropenia, leukopenia, thrombocytopenia, lymphopenia, hypoalbuminemia, aspartate aminotransferase elevation, glutamyl transpeptidase elevation, anemia, and alanine aminotransferase elevation.

**Conclusions:**

Despite the rate of PEPI score 0 was not high, tucidinostat plus exemestane as a neoadjuvant therapy might be well tolerated and showed promising clinical responses in patients with early hormone receptor-positive, HER2-negative breast cancer. To clarify the safety and efficacy of this strategy, further investigation is warranted.

**Clinical Trial Registration:**

ChiCTR2100046678.

## Teaser

This study assessed the efficacy and safety of tucidinostat plus exemestane as a neoadjuvant strategy in early-stage breast cancer.

**Implications for Practice**

Despite the rate of preoperative endocrine prognostic index score 0 was not high, tucidinostat plus exemestane as a neoadjuvant therapy might be well tolerated and showed promising clinical responses in patients with early hormone receptor-positive, human epidermal growth factor receptor 2-negative breast cancer. The findings of this study suggested that tucidinostat plus exemestane could represent an alternative treatment modality and provide a possible therapeutic opportunity for these patients.

## Introduction

Breast cancer is the most prevalent cancer in women worldwide,^1^ with hormone receptor-positive breast cancer constituting over 70% of cases. Endocrine therapy serves as a standard treatment for hormone receptor-positive breast cancer. In recent years, neoadjuvant therapy has demonstrated its ability to downstage tumors, providing patients with enhanced surgical options and survival benefits.^2^ However, the response to neoadjuvant chemotherapy (NCT) varies among different molecular subtypes of breast cancer,^3^ with luminal breast cancer exhibiting a lower pathological complete response (pCR) rate of only 10%-20%.^4^

While neoadjuvant endocrine therapy is considered a viable clinical treatment strategy,^5^ the process may lead to primary or secondary endocrine resistance. To overcome resistance and improve efficacy, combining endocrine therapy with other drugs has become a common clinical strategy. Presently, ongoing clinical trials focusing on neoadjuvant endocrine therapy predominantly involve CDK4/6 inhibitors, such as the neoadjuvant-palbociclib (NeoPal) and NeoPalAna studies.^6^ Nevertheless, the use of CDK4/6 inhibitors has been associated with a notable increase in adverse effects, including diarrhea, nausea, neutropenia, leukopenia, thrombocytopenia, and fatigue.^7,8^

Histone deacetylase (HDAC) inhibitors have emerged as a novel strategy for cancer treatment due to their regulation of epigenetic changes, which play a critical role in tumor development, immune regulation, drug resistance, and recurrence.^9,10^ One key advantage of HDAC is their relatively lower rate of side effects. Moreover, most side effects associated with HDAC inhibitors are primarily observed with pan-HDAC inhibitors,^11^ indicating that subtype-selective HDAC inhibitor may have fewer adverse effects. Tucidinostat, an oral subtype-selective HDAC inhibitor, received approval from the National Medical Products Administration in China in November 2019 for use in combination with aromatase inhibitors in postmenopausal patients with locally advanced or metastatic breast cancer who have experienced relapse or progression on endocrine therapy. Jiang et al^12^ evaluated the efficacy and safety of tucidinostat plus exemestane in patients with hormone receptor-positive, human epidermal growth factor receptor 2 (HER2)-negative advanced breast cancer. It has been reported that exemestane could be a safe and effective drug for hormone receptor-positive and HER2-negative metastatic breast cancer.^13^ Given the potential of tucidinostat in overcoming endocrine resistance, the combination of HDAC inhibitors with endocrine therapy holds promise for achieving satisfactory results. Therefore, this study aimed to investigate the efficacy and safety of tucidinostat plus exemestane as a neoadjuvant strategy in early-stage breast cancer with hormone receptor-positive and HER2-negative (ChiCTR2100046678).

## Materials and Methods

### Study Design and Patients

This prospective, single-center, open-labeled, single-arm phase II study enrolled patients with stage II-III breast cancer with hormone receptor-positive and HER2-negative who were admitted to Tianjin Medical University Cancer Institute and Hospital between January and November 2021. Eligible patients were (1) aged > 35 years; (2) with histologically confirmed invasive breast cancer with estrogen receptor (ER)-positive (>50%) and HER2-negative (ASCO/CAP); (3) TNM clinical stage II-III (the maximum diameter of the tumor was >2 cm); (4) had an Eastern Cooperative Oncology Group (ECOG) score of 0-2; (5) previous untreated for breast cancer; (6) with absolute neutrophil count ≥ 1.5 × 10^9^/L, platelets ≥ 100 × 10^9^/L, and hemoglobin ≥ 90 g/L; and (7) voluntarily participated in this clinical trial, signed written informed consent. Exclusion criteria: (1) no measurable lesion, such as pleural or pericardial effusion, ascites, etc.; (2) had undergone major surgical procedures or significant trauma within 4 weeks prior to enrollment, or anticipated that the patient would undergo major surgical treatment (non-breast cancer related); (3) previous treatment with HDAC inhibitors (including romidepsin, vorinostat, belinostat, panobinostat, and entinostat); (4) those with known history of allergy to the components of the drugs of this protocol; (5) have a history of immunodeficiency, including a positive HIV test, or have other acquired, congenital immunodeficiency diseases, or have a history of organ transplantation; (6) uncontrolled significant cardiovascular disease, history of clinically significant QT interval prolongation, or QTc interval > 450 ms at screening; (7) abnormal liver function [total bilirubin > 1.5 times upper limit of normal; ALT/AST > 2.5 times the upper limit of normal] and abnormal renal function (serum creatinine > 1.5 times the upper limit of normal); (8) a positive pregnancy test at baseline in a pregnant, lactating female patient or a female of childbearing potential, or reproductive age subjects who are unwilling to take effective contraception during study participation and at least 8 weeks after the last dose; (9) serious harms to patient safety, or concomitant diseases affecting patient completion of the study (such as severe hypertension, diabetes mellitus, thyroid disease, and active infection) as judged by the investigator; (10) previous clear history of neurological or psychiatric disorders, including epilepsy or dementia; and (11) not judged by the investigator to be suitable for participation in this study. This study was approved by the Ethics committee of Tianjin Medical University Cancer Institute and Hospital. Written informed consents were obtained from all patients.

### Intervention

Prior to study entry, all patients underwent breast and axillary disease assessment using cone-beam breast computed tomography (CBBCT). Additionally, patients underwent an evaluation of their ECOG performance status, core biopsy, complete blood cell count, serum chemistry, electrocardiogram, and chest X-ray. Eligible patients received oral tucidinostat at a dose of 30 mg twice weekly in combination with daily oral exemestane at a dose of 25 mg for a duration of up to 24 weeks. Premenopausal patients also received subcutaneous goserelin at a dose of 3.6 mg every 28 days for a total of 6 doses. Following neoadjuvant treatment, patients underwent either mastectomy or conservative surgery along with axillary lymph node dissection or sentinel lymph node biopsy. The evaluation of patients took place after the completion of neoadjuvant therapy. Surgery was scheduled 3 weeks after the final administration of neoadjuvant therapy for patients eligible for the procedure, and breast-conserving surgery (BCS) or modified radical mastectomy was performed on those who were both willing and qualified for the procedure (Supplementary Fig. S1). The postoperative radiotherapy and its protocol were determined by the investigator. There was no predefined postoperative adjuvant treatment plan, allowing for flexibility in postoperative systemic treatment. In cases where patients were considered inoperable by the investigator, the subsequent treatment plan was a collaborative decision between the investigator and the patient. Prior to participating in the study, all patients provided written informed consent.

### Measurements and Endpoints

The primary endpoint was patients who achieved a preoperative endocrine prognostic index (PEPI) score of 0. The PEPI score system is a classical prognostic score system, which includes tumor size, lymph node status, Ki67 expression level, and ER status. A multivariable Cox proportional hazard regression model was used to evaluate the independent prognostic relevance of each factor, namely, pathological tumor size, pathological node status, clinical response, surgical specimen ER status, histological grade, and the Ki67 level. Hazard ratio (HR) estimates of each factor in the final multivariable model were used to construct a score, the PEPI, for risk of relapse-free survival (RFS) and breast cancer–specific survival (BCSS). The PEPI score was derived as an arithmetic sum of risk points weighted by the size of the HR assigned to each statistically significant factor (16). RFS was defined as the interval between random assignment to treatment and the earliest subsequent breast cancer event. BCSS was defined as the interval between random assignment and the date of death after breast cancer relapse.

The secondary endpoints included complete cell cycle arrest (CCCA, defined as Ki67 < 2.7%, indicating that the cell cycle progression is completely suppressed), pathological complete response (pCR, indicating no tumor can be detected by pathological examination) rate, objective response rate (ORR, the proportion of patients who achieve a specified reduction in tumor volume and maintain it for the minimum duration, including complete response [CR], partial response [PR], and stable disease [SD]) assessed by radiography, disease control rate (DCR, the percentage of patients who achieved disease response and stability after treatment in the total number of evaluable patients), BCS, as well as assessing safety and tolerability. Clinical response was evaluated according to the RECIST criteria^14^ every 6 weeks using CBBCT. The pCR was assessed at the time of surgery, employing the Miller and Payne criteria.^15^ Adverse events (AEs) were graded following the National Cancer Institute Common Terminology Criteria for Adverse Events version 4.03, and the most severe grade experienced by each patient was reported.

### Statistical Analysis

All analyses were performed using SPSS 22.1 (IBM Corp., Armonk, NY, USA). Continuous data were expressed as mean ±  SD and compared by *t* test. Categorical data were expressed as *n* (%), and compared by the Chi-square test. The primary analysis focused on determining the proportion of patients achieving a PEPI 0 patients in this study, and 95% CIs were calculated for this proportion. The primary endpoint was evaluated per protocol set. Secondary endpoints were assessed using descriptive statistical methods, and the results were presented along with their 95% CIs based on per protocol set. Safety analysis was performed on the safety set, which included all patients who received the treatment dose. Two-sided *P* < .05 was considered statistical significance.

## Results

The study enrolled a total of 22 women diagnosed with stage II-III breast cancer, out of whom 2 patients discontinued the study treatment due to noncompliance. Finally, 20 patients successfully completed the treatment regimen and subsequently underwent surgery. Among them, 5 (25%) underwent BCS, while 15 received modified radical mastectomy (Supplementary Fig. S2). The median age of the patients was 52.5 (range from 36 to 75) years, and 17 (85%) of them were diagnosed with invasive ductal carcinoma and all patients exhibited progesterone receptor-positive tumors (Table 1).

**Table 1.** Baseline characteristics.

Variables	Tucidinostat + exemestane (*n* = 20)
Median age (range)	52.5 (36-75)
Menopausal status	
Premenopausal	9 (45.0%)
Postmenopausal	11(55.0%)
ECOG score	
0	18 (90.0%)
1	2 (10.0%)
2	0
Tumor size (CBBCT^1^)	
T1	0
T2	16 (80.0%)
T3	4 (20.0%)
Lymph node status^2^	
N0	10 (50.0%)
N1	10 (50.0%)
N2	0
N3	0
Clinical stage	
IIA	8 (40%)
IIB	11 (55%)
IIIA	1 (5%)
IIIB/C	0
Tumor grade	
1	1 (5%)
2	18 (90%)
3	1 (5%)
Histology	
Ductal carcinoma	17 (85%)
Others	3 (15%)
Estrogen receptor expression	
≥ 50%	20 (100%)
< 50%	0
Progesterone receptor expression	
≥10%	20 (100%)
<10%	0
Ki67 expression	
≥ 20%	9 (45%)
< 20%	11 (55%)
Luminal type	
Luminal A	9 (45%)
Luminal B	11 (55%)
EGFR	
≥1	3 (15%)
<1	17 (85%)

^1^Baseline cone-beam breast computed tomography measure.

^2^As defined by cone-beam breast computed tomography and needle aspiration.

Abbreviations: ECOG, Eastern Cooperative Oncology Group; CBBCT, cone-beam breast computed tomography; ER, estrogen receptors; PR, progesterone receptor.

**Figure 1.**
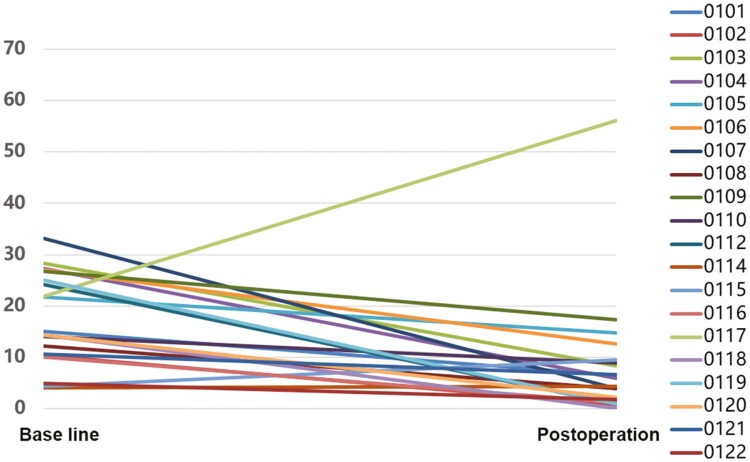
Change of Ki67 expression. Ki67 suppression from baseline to surgery can be observed in 17 patients.

In all patients, 3 (15%) patients in both BCSS (*n* = 20) and RFS (*n* = 20) achieved PEPI score of 0, while 10 (50%) of whom achieved PEPI score between 1 and 3, and 7 (35%) of whom achieved PEPI score of 4 or above (Table 2). In addition, CCCA was observed in 7 (35%). Radiologic ORR was observed in 10 (50%), including 1 (5%) achieved CR, 9 (45%) achieved PR, and DCR was achieved in all 20 (100%) patients (Fig. 1). pCR was observed in 1 (5%) patient, and 7 (35%) patients performed BCS (Table 3).

**Table 2.** Primary endpoint.

PEPI score, *n* (%)	RFS	BCSS
0	3 (15%)	3 (15%)
1-3	10 (50%)	10 (50%)
4 or above	7 (35%)	7 (35%)

Abbreviations: PEPI, preoperative endocrine prognostic index; RFS, relapse-free survival; BCSS, breast cancer-specific survival.

**Table 3.** Secondary endpoints.

Index	*N* (%)
CCCA	7 (35)
Radiologic ORR	10 (50)
CR	1 (5)
PR	9 (45)
SD	10 (50)
DCR	20 (100)
pCR	1 (5)
BCS	5 (25)

Abbreviations: CCCA, complete cell cycle arrest; CR, complete response; PR, partial response; SD, stable disease; ORR, objective response rate; DCR, disease control rate; pCR, pathological complete response; BCS, breast-conserving surgery.

Additionally, in a subgroup of patients with Ki67 expression < 20% (*n* = 11), 2 (18.2%) of them in both BCSS and RFS achieved PEPI score of 0, 6 (54.5%) achieved PEPI score between 1 and 3, and 3 (27.3%) achieved PEPI score of 4 or above. Among the subgroup of patients with Ki67 expression ≥ 20% (*n* = 9), 1 (11.1%) of them in both BCSS and RFS achieved PEPI score of 0, 4 (44.4%) achieved PEPI score between 1 and 3, and 4 (44.4%) achieved PEPI score of 4 or above. In a subgroup of premenopausal patients (*n* = 9), 1 (11.1%) of them in both BCSS and RFS achieved PEPI score of 0, 4 (44.4%) achieved PEPI score between 1 and 3, and 4 (44.4%) achieved PEPI score of 4 or above. Among the subgroup of postmenopausal patients (*n* = 11), 2 (18.2%) of them in both BCSS and RFS achieved PEPI score of 0, 6 (54.5%) achieved PEPI score between 1 and 3, and 3 (27.3%) achieved PEPI score of 4 or above (Supplementary Table S1). Additionally, the radiologic ORR in the subgroup of patients with Ki67 > 20% and those with Ki67 ≤ 20% were 45.45% and 55.56%, respectively. While, in the subgroup of premenopausal and postmenopausal patients were 55.56% and 45.45%, respectively (Supplementary Table S2).

**Figure 2.**
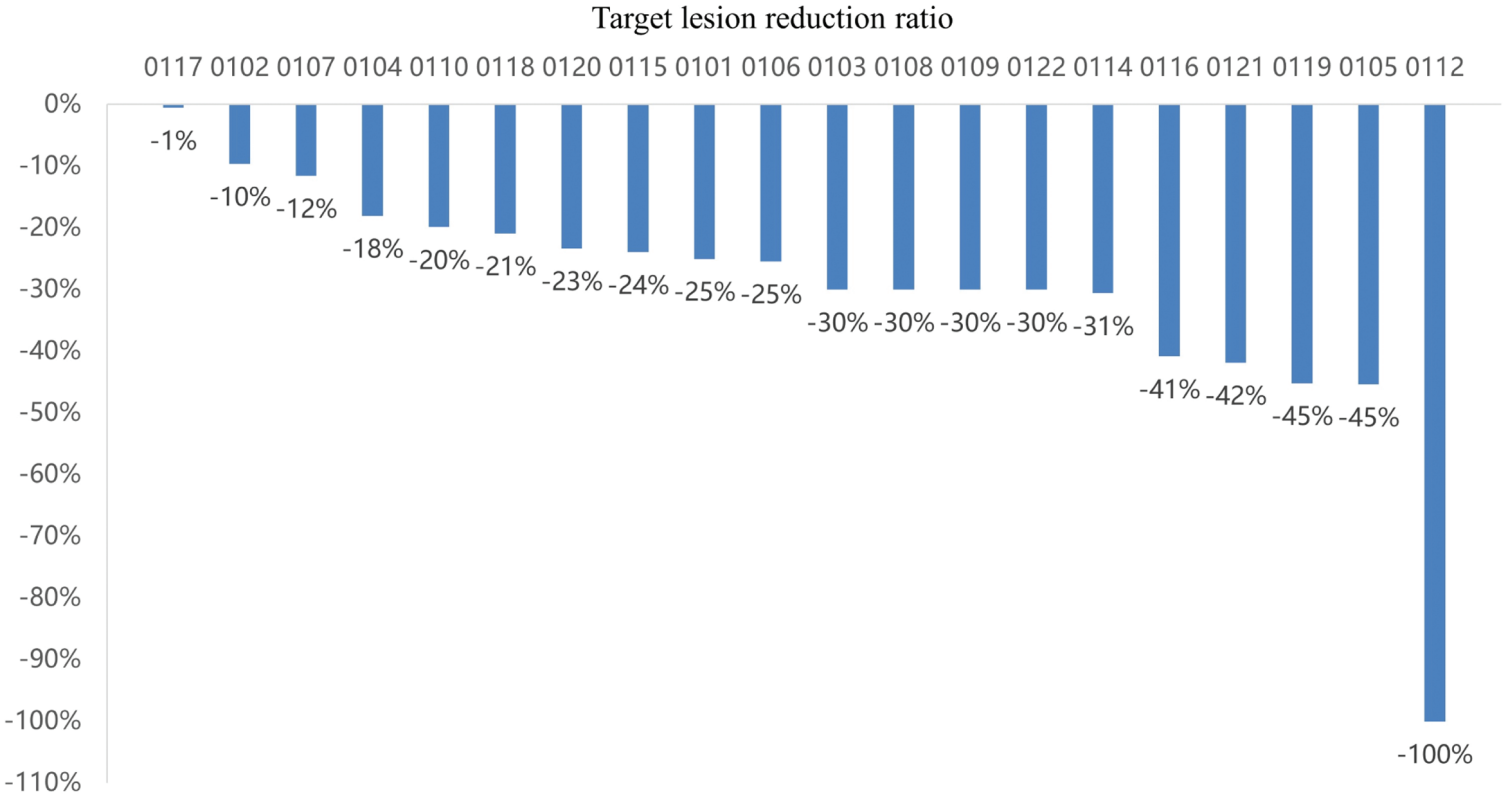
Target lesion reduction ratio. Efficacy evaluation: CR in 1 case, PR in 9 cases, SD in 10 cases, ORR = 50%, DCR = 100%.

In the subgroup of patients with Ki67 > 20%, 9 (81.8%) of them were demonstrated reduced Ki67 expression, with a Ki67 change ratio of −73.4%, and a CCCA rate of 45.5%. Among the subgroup of patients with Ki67 ≤ 20%, 8 (88.9%) of them showed reduced Ki67 expression, with a Ki67 change ratio of −73.7%, and a CCCA rate of 22.2%. In the subgroup of premenopausal patients, 7 (77.8%) of them exhibited a reduction in Ki67 expression, with a Ki67 change ratio of −72.6%, and a CCCA rate of 22.2%. Among the subgroup of postmenopausal patients, 10 (90.9%) of them exhibited a reduction in Ki67 expression, with a Ki67 change ratio of −74.2%, and a CCCA rate of 45.5% (Supplementary Table S3). The Ki67 suppression from baseline to surgery exhibited in 85% patients, and CCCA at the time of surgery was observed in 35% of patients (Fig. 2).

Safety and tolerability evaluation showed that all patients experienced at least one treatment-emergent adverse event during the course of the study, including neutropenia (95%), leukopenia (85%), thrombocytopenia (65%), lymphopenia (55%), hypoalbuminemia (50%), aspartate aminotransferase elevation (35%), glutamyl transpeptidase elevation (30%), anemia (25%), and alanine aminotransferase elevation (25%).

## Discussion

Tucidinostat (formerly known as chidamide) is an oral benzamide class of HDAC inhibitor with subtype specificity for inhibition of HDAC1, HDAC2, HDAC3, and HDAC10.^16^ Basic studies have shown that benzamide subtype-selective HDAC inhibitors, including tucidinostat and entinostat, enhance tumor immune surveillance by activating natural killer cell- and antigen-specific cytotoxic T-cell-mediated cellular antitumor immunity. This distinguishes subtype-selective drugs from other nonselective HDAC inhibitors.^16-18^ Tucidinostat can inhibit the bypass growth factor signaling pathway and increase the lysine acetylation level at some sites of ER, directly inhibit the ligand-dependent activation pathway, and restore the sensitivity of anti-estrogen drugs.^19^

This clinical trial showed that, despite the rate of PEPI scored 0 was not high, tucidinostat plus exemestane as a neoadjuvant therapy might be well tolerated and showed promising clinical responses in patients with early hormone receptor-positive, patients with HER2-negative breast cancer. The findings of this study suggested that tucidinostat plus exemestane could represent an alternative treatment modality and provide a possible therapeutic opportunity for these patients.

The PEPI score was used as the primary endpoint in this study, which includes tumor size, lymph node status, Ki67 expression level, and ER status.^20^ The P024 study demonstrated varying long-term recurrence risks in different PEPI score subgroups,^20^ and this finding was subsequently confirmed in the prospective study IMPACT.^21^ The findings showed that 15% of patients (3/20) achieved PEPI 0 scores, suggesting that the combination of tucidinostat with exemestane might bring a potential long-term beneficial effect for those patients. The Z1031 study demonstrated that the subgroup receiving postoperative adjuvant chemotherapy had a significantly lower recurrence risk compared to the PEPI non-0 subgroup (*P* = .0221), with only a 2.9% recurrence risk at 5 years.^22^ Therefore, for the PEPI = 0 subgroup after neoadjuvant endocrine therapy, postoperative adjuvant chemotherapy could be considered unnecessary.

Moreover, findings from the IMPACT study confirmed a significant association between high Ki67 expression after 2 weeks of neoadjuvant therapy and low RFS.^21^ The Z1031B study showed that Ki67 > 10% during 2-4 weeks of neoadjuvant endocrine therapy significantly increased the risk of recurrence.^22^ As a result, subsequent neoadjuvant studies have incorporated PEPI score and Ki67-based endpoints for evaluation. The findings in this present study showed that significant Ki67 suppression from baseline to surgery was observed in the majority of patients, with decreased Ki67 expression observed in both premenopausal and postmenopausal populations, as well as in different subgroups based on Ki67 expression. And the lack of significant differences among subgroups suggests an improved therapeutic effect.

The majority of existing studies^22^ on neoadjuvant endocrine therapy have been focused on postmenopausal patients, leaving the efficacy in premenopausal patients unclear. In this clinical trial, the subgroup analysis of premenopausal and postmenopausal populations showed no difference in RFS and BCSS, suggesting that the combination of tucidinostat with exemestane has similar efficacy in both groups of patients. Therefore, the clinical implications of this study are significant.

The findings of this study reveal that 5% of patients achieved pCR, while 35% exhibited CCCA at the time of surgery (Ki67 ≤ 2.7%). Similar results have been demonstrated in other studies involving the use of CDK4/6 inhibitors in neoadjuvant endocrine therapy, indicating a noteworthy improvement in CCCA rates. For instance, a phase II trial comparing CCCA with anastrozole plus palbociclib versus anastrozole monotherapy showed a significant improvement in CCCA rates.^23^ The neoMONARCH study, which used a combination of anastrozole and abesilib, significantly improved the CCCA rate when compared to anastrozole alone.^24^ Johnston et al’s^25^ investigation demonstrated that palbociclib combined with letrozole significantly inhibited tumor cell proliferation. Although the application of CDK4/6 inhibitors in NET has been effective in enhancing the inhibition of CCCA rates, the performance of pCR caused by CDK4/6 was poor when compared with the combination of HDAC inhibitor tucidinostat and exemestane, with only 3.7% of the patients in NeoMONARCH achieving pCR,^24^ and an even lower proportion of patients reaching pCR in the N007 study.^26^ Besides, no pCR on NeoPalAna study was reported.^23^

However, the evaluation of using pCR alone is limited, and the residual cancer burden (RCB) score, which is defined as a continuous index combining pathologic measurements of primary tumor (size and cellularity) and nodal metastases (number and size) for prediction of distant RFS, represents the latest method for grading pathological response.^27^ In the NeoPAL trial, pCR (RCB score 0 to 1) was achieved in 7.7% of the letrozole plus palbociclib group and 15.7% of the NCT.^28^

In our study, the breast preservation rate of patients was 25%, which is lower than that reported in studies conducted in Western countries but consistent with the rate among Chinese (12%-24%).^29-32^ This could be attributed to disparities in the stage of diagnosis and breast volume between China and Europe or the USA. European and American patients are often diagnosed at an earlier stage^33^ with higher volume of breast^34^ compared to their Chinese counterparts, resulting in some Chinese patients missing out on the opportunity for breast conservation. In our study, only 80% of patients were initially diagnosed with stage T1-2, while more than 90% of patients in the NeoPal trial had tumors <5 cm.^28^ Considering the differences of breast volume, although hard to compare, it is understandable that the breast preservation rate in our study was lower than in NeoPal. Economic factors also significantly impact patient decision-making processes as radiotherapy costs impose financial burdens on individuals seeking treatment.^30^ Furthermore, disparities between urban and rural areas, educational attainment levels, and the overall quality of medical institutions are all influential factors impacting breast preservation rates.^35^ Moreover, due to uneven distribution of medical resources, high transportation costs coupled with time constraints discourage some individuals from pursuing breast-conserving therapy outside their local area hospitals.

This study used CBBCT as the evaluation method to assess therapeutic effect, setting it apart from most previous studies on neoadjuvant endocrine therapy that used ultrasound or MRI. Although MRI is recognized as the most accurate imaging examination method,^36,37^ it fails to show calcification^38,39^ and can often be costly and time-consuming, potentially excluding patients who cannot tolerate MRI.^40^ CBBCT, a novel breast CT imaging equipment, uses a semi-conical X-ray beam to radiate the breast, a plate detector to collect the image information, and computer-based 3-dimensional reconstruction for a comprehensive 3-dimensional breast image.^41,42^ Existing studies have shown that breast CT surpasses MRI in the accuracy of tumor residual assessment in luminal breast cancer.^43,44^

This study observed ORR, which showed no difference in different subgroups and was similar to NCT, demonstrating a good safety profile. None of the 20 subjects experienced grade 4 adverse reactions, and only three grade 3 hematological adverse reactions were reported, specifically neutropenia, leukopenia, and thrombocytopenia. Additionally, one grade 3 nonhematological adverse reaction, pneumonia, was recorded as a serious adverse event. The NeoPAL trial revealed no significant difference in progression-free survival and invasive disease-free survival,^28^ but the incidence of AEs in the chemotherapy group was almost twice that in the letrozole plus palbociclib group. A meta-analysis of 20 studies showed that neoadjuvant endocrine therapy using aromatase inhibitor, when compared with NCT, showed no significant differences in ORR, imaging response rate, pCR, and BCS, but the neoadjuvant endocrine therapy showed better safety profiles.^45^

This study has several limitations. Firstly, it was a single-center trial, which limited the generalizability or external validity of the findings. Additionally, the sample size was relatively small. Consequently, larger-scale and multicenter studies are needed in the near future to validate the results. Furthermore, the follow-up period was relatively short, necessitating further investigation to determine whether tucidinostat plus exemestane can lead to improvements in long-term prognosis.

In summary, despite the low proportion of PEPI scores of 0, tucidinostat plus exemestane may be well tolerated as neoadjuvant therapy in patients with early hormone receptor-positive, HER2-negative breast cancer. However, due to the small sample size of this study, although the combination therapy showed some clinical efficacy, larger-scale clinical trials are needed and the favorable benefit-risk profile of the combination therapy needs to be further evaluated.

## Supplementary Material

oyae033_suppl_Supplementary_Material

oyae033_suppl_Supplementary_Data

## Data Availability

All data generated or analyzed during this study are included in this published article.
