# Supplementary material for: Tucidinostat Plus Exemestane as a Neoadjuvant in Early-Stage, Hormone Receptor-Positive, Human Epidermal Growth Factor Receptor 2-Negative Breast Cancer
Source: Oncologist. 2024 Mar 9;29(6):e763–70. doi: 10.1093/oncolo/oyae033 (PMC11144976; doi:10.1093/oncolo/oyae033)
Supplement: oyae033_suppl_Supplementary_Material [file oyae033_suppl_supplementary_material.docx]

**Supplementary Tables and Figures**

**
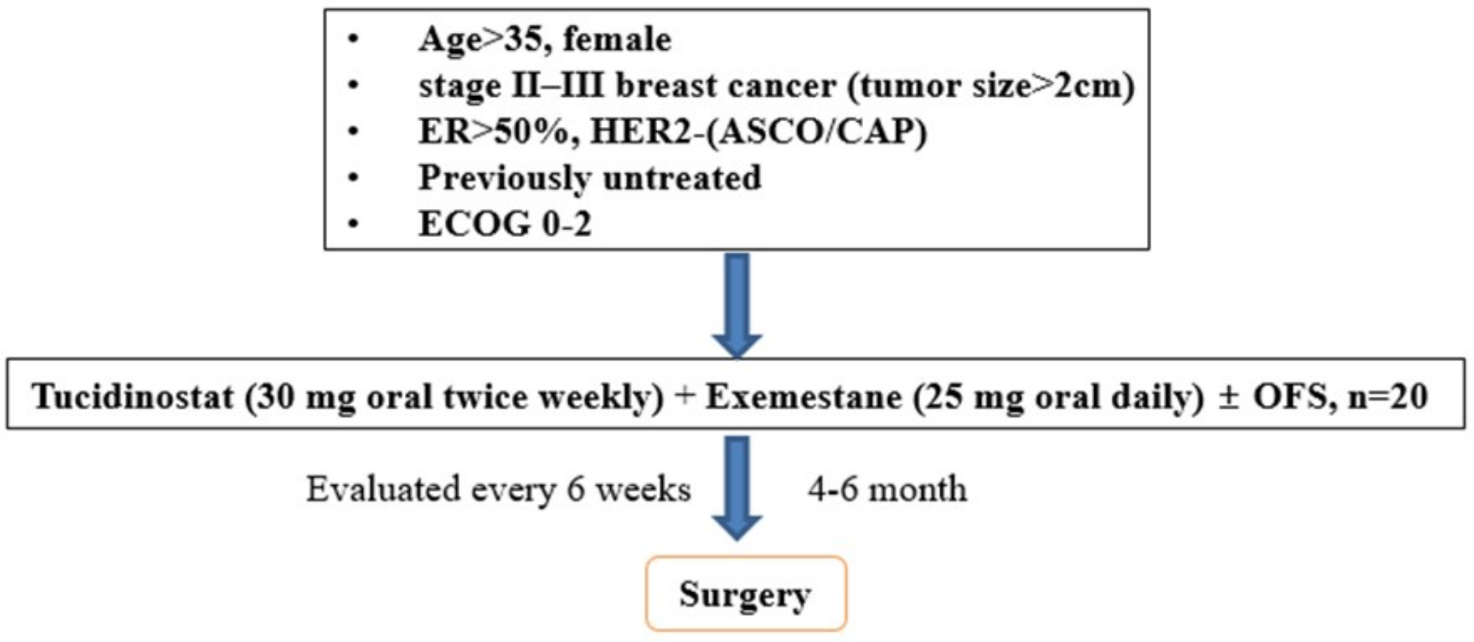
**

**Fig. S1** Study design. Patients with HR-positive, HER2-negative, and node-positive, stage II-III breast cancer were enrolled. Eligible patients received 30 mg oral tucidinostat twice weekly in combination with 25 mg oral exemestane daily for up to a total of 24 weeks.


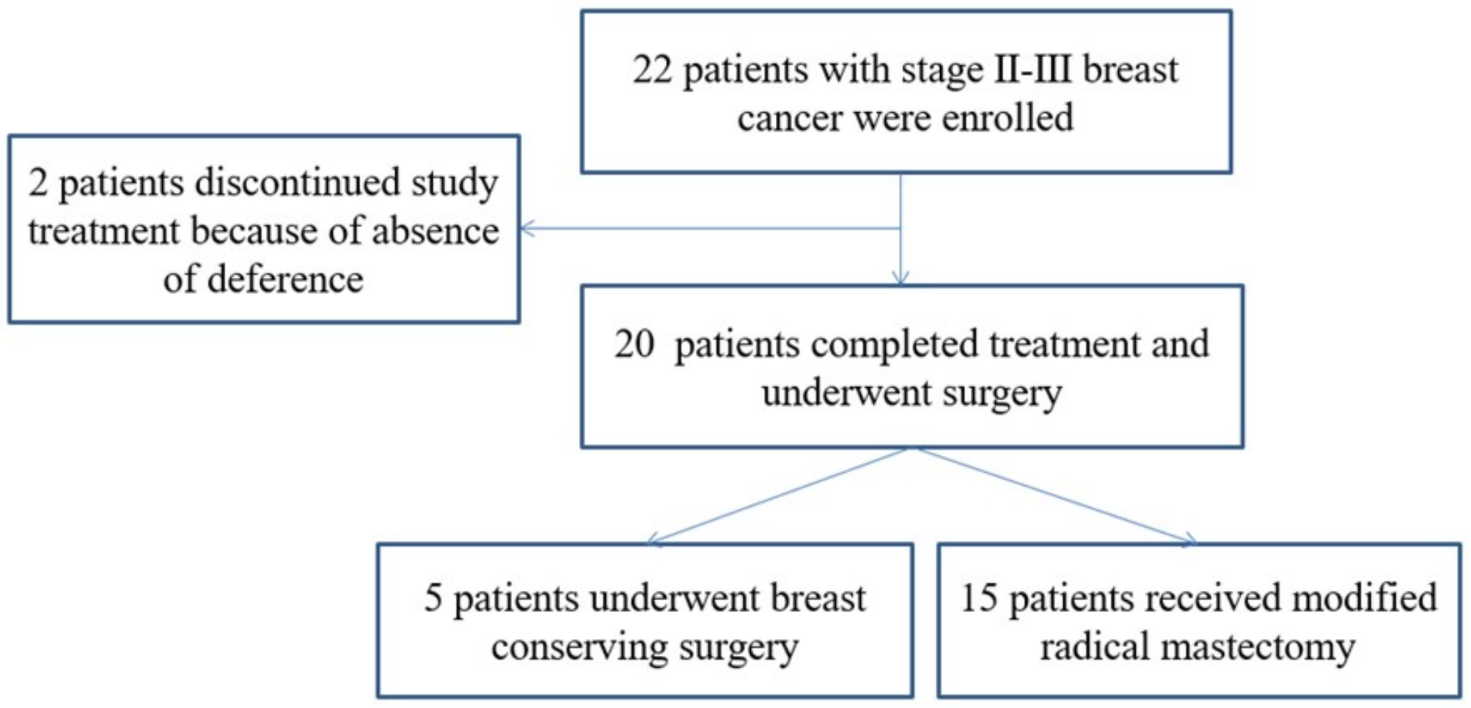


**Fig.S2** Trail profile. Twenty-two women with stage II-III breast cancer were enrolled, two patients discontinued study treatment because of absence of deference. Five patients who underwent breast conserving surgery.

**Table S1**. Subgroup analysis for PEPI and radiologic ORR.

| PEPI score | RFS | | | BCSS | | |
| --- | --- | --- | --- | --- | --- | --- |
|  | Ki67<20% (n=11) | Ki67≥20% (n=9) | P | Ki67<20% (n=11) | Ki67≥20% (n=9) | P |
| PEPI=0 | 2 (18.2%) | 1 (11.1%) | 1 | 2 (18.2%) | 1 (11.1%) | 1 |
| PEPI=1-3 | 6 (54.5%) | 4 (44.4%) | 1 | 6 (54.5%) | 4 (44.4%) | 1 |
| PEPI≥4 | 3 (27.3%) | 4 (44.4%) | 0.6 | 3 (27.3%) | 4 (44.4%) | 0.6 |
|  | Premenopause (n=9) | Postmenopausal (n=11) | P | Premenopause (n=9) | Postmenopausal (n=11) | P |
| PEPI=0 | 1 (11.1%) | 2 (18.2%) | 1 | 1 (11.1%) | 2 (18.2%) | 1 |
| PEPI=1-3 | 4 (44.4%) | 6 (54.5%) | 1 | 4 (44.4%) | 6 (54.5%) | 1 |
| PEPI≥4 | 4 (44.4%) | 3 (27.3%) | 0.6 | 4 (44.4%) | 3 (27.3%) | 0.6 |

RFS: relapse-free survival; BCSS: breast cancer specific survival; PEPI, preoperative endocrine prognostic index;

**Table S2**. Subgroup analysis for radiologic ORR.

|  | Ki67 stratification | | | Menopausal state stratification | | |
| --- | --- | --- | --- | --- | --- | --- |
|  | Ki67<20% (n=11) | Ki67≥20% (n=9) | P | Premenopause (n=9) | Postmenopausal (n=11) | P |
| Total | 55.00% | 45.00% | >0.999 | 45.00% | 55.00% | >0.999 |
| Radiologic ORR | 45.45% | 55.56% | >0.999 | 55.56% | 45.45% | >0.999 |

ORR, objective response rate.

**Table S3**. Comparison of Ki67 changes, CCCA, and pCR.

| Index | Ki67 stratification | | | Menopausal state stratification | | |
| --- | --- | --- | --- | --- | --- | --- |
|  | Ki67<20 (n=11) | Ki67≥20 (n=9) | P | Premenopause (n=9) | Postmenopausal (n=11) | P |
| Ki67 median value % (baseline) | 10.7 | 26.8 | <0.001 | 21.9 | 14.1 | 0.31 |
| Ki67 median value % (sugery) | 4 | 8.3 | 0.13 | 6.5 | 4 | 0.003 |
| Ki67 increased by % (n/N) | 2 (18.2%) | 11.1(1/9) | >0.999 | 2 (22.2%) | 1 (9.1%) | 0.57 |
| Ki67 reduced % (n/N) | 9 (81.8%) | 88.9(8/9) | >0.999 | 7 (77.8%) | 10 (90.9%) | 0.57 |
| Ki67 change ratio % | -73.4 | -73.7 | 0.94 | -72.6 | -74.2 | 0.62 |
| CCCA% (n/N) | 5 (45.5%) | 2 (22.2%) | 0.37 | 2 (22.2%) | 5 (45.5%) | 0.37 |
| pCR% (n/N) | 0 | 11.1(1/9) |  | 11.1(1/9) | 0 |  |

CCCA, complete cell cycle arrest; pCR, pathological complete remission.
