# Supplementary material for: Tucidinostat Plus Exemestane as a Neoadjuvant in Early-Stage, Hormone Receptor-Positive, Human Epidermal Growth Factor Receptor 2-Negative Breast Cancer
Source: Oncologist. 2024 Mar 9;29(6):e763–70. doi: 10.1093/oncolo/oyae033 (PMC11144976; doi:10.1093/oncolo/oyae033)
Supplement: oyae033_suppl_Supplementary_Data [file oyae033_suppl_supplementary_data.docx]

**Cedarbin, amine combined with exemestane in neoadjuvant treatment of early HR positive breast cancer**

**testing program**

declaration of secrecy

This protocol is only provided to the investigator, the ethics committee and the relevant regulatory authorities, and the ownership of the drug-related information involved belongs to the drug partner. Without the written consent of the partner, do not give any explanation without the necessary explanation to the subjects who may participate in the clinical trial.

# scheme

| research topic | Cedarbin, amine combined with exemestane in neoadjuvant treatment of early HR positive breast cancer |
| --- | --- |
| Research nature | Open, single-center, cohort, exploratory study |
| subject investigated | Initial diagnosis was stage II-III (maximum tumor diameter> 2cm), ER expression> 50%, HER 2 negative, and previously untreated breast cancer patients |
| purpose of research | To evaluate the efficacy and safety of sitabenamine combined with exemestane in neoadjuvant treatment for stage II-III HR positive breast cancer; |
| Number of patients enrolled | 20 Cases |
| research design | The experimental design route is given as follows  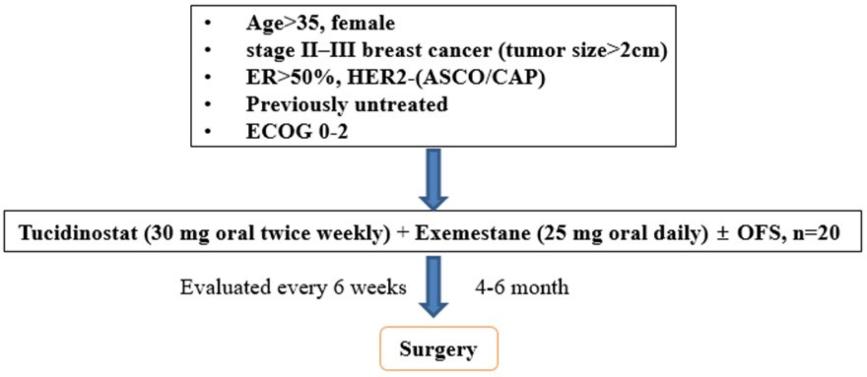 |
| research on drug | Cedardendamine tablets, 5mg / tablet, 6 tablets / time, 2 times / week  exemestane tablets, 25mg / tablet, 1 tablet / time, 1 time / day  OFS medication (used in premenopausal patients only) |
| The end of the study | **Main end point:**   - Show the proportion of the PEPI score =0 after surgery   **Secondary end point:**   - Complete cell cycle arrest (CCCA: Ki-672.7%) - Pathological complete response rate (pCR: ypT 0 / is ypN0) - Clinical Objective Response Rate (ORR) - Disease control rate (DCR), - Breastproof surgery rate - Event-free survival (EFS) - Overall survival (OS) - Adverse Event (AE) |
| Sample size determination | This study is a small sample, exploratory clinical study, according to the clinical data analysis requirements. |
| Enrollment criteria  Enrollment criteria | **Patients must meet all of the following criteria to be enrolled:**  1. Age> 35 years old, female;  2. Histologically or cytologically confirmed invasive breast cancer with ER expression> 50% and HER 2 negative *;  3. TNM clinical stage II-III (maximum tumor diameter> 2cm);  4. Previously untreated for breast cancer;  5. At least one measurable primary lesion was present (according to the RECIST v1.1 standard) before enrollment;  6. The ECOG score is 0-2 points;  7. Absolute value of neutrophils 1.5109 / L, platelet 100109 / L, and hemoglobin 90g / L;  8. Voluntary participated in this clinical trial and signed a written informed consent  * HER 2 negative was defined as: ① IHC 1 + / IHC 0; ② IHC2 +: FISH- (HER-2 / CEP17 ratio <2.0 and mean HER-2 copy number / cell <4.0) or investigator judged HER-2 negative; |
| Exclusion criteria  Exclusion criteria | **Patients will not be selected if they meet any of the following conditions:**  1. No measurable lesions, such as pleural or pericardial exudate, ascites, etc.;  2. A major surgery or significant trauma within 4 weeks before enrollment, or the patient is expected to receive major surgery (non-breast cancer related);  3. Previous treatment with HDAC inhibitors (including roidine, vorinostat, belisostat, palbidostat, entinat, etc.);  4. Patients with a known allergy history of drug components of this regimen;  5. History of immunodeficiency, including positive HIV test, or other acquired, congenital immunodeficiency diseases, or history of organ transplantation;  6. Ununcontrolled cardiovascular disease; history of clinically significant QT interval extension, or QTc interval> 450 ms;  7. Abnormal liver function [1.5 times the upper limit of total bilirubin; 2.5 times the upper limit of ALT / AST in patients without liver metastasis, 5 times of ALT / AST in patients with liver metastasis], abnormal renal function (1.5 times the upper limit of serum creatinine> normal);  8. Positive baseline pregnancy test for pregnant or lactating women or fertility women; or subjects of childbearing age who are unwilling to use effective contraception during the study and for at least 8 weeks after the last dose;  9. According to the judgment of the investigator, there are serious concomitant diseases (such as severe hypertension, diabetes, thyroid disease, active infection, etc.) affecting the completion of the study;  10. Prior history of neurological or psychiatric disorders, including epilepsy or dementia;  11. The investigator was judged unfit to attend the investigator. |
| Termination standard | The subject must withdraw / terminate treatment if one or more of the following conditions occurs:  1. Patients are requested to withdraw from the study;  2. Disease progression or death of patients;  3. Patients have severe toxicity and cannot accept the study protocol;  4. The investigator considers the termination of the study necessary;  5. Patients seriously did not cooperate with the study. |
| The study period | The preset is 15 months and the first 10 months is the subject enrollment period. |
| statistical treatment  statistical treatment | The test population  This trial uses an adjusted intention-to-treat (mITT) population and a safety population. The intent-to-treat population included all subjects who signed informed consent and completed neoadjuvant therapy, patients who did not complete neoadjuvant therapy were excluded from the analysis set; patients who completed neoadjuvant therapy but did not undergo surgery were considered PEPI non-0. The safety population was those receiving at least 1 medication. Efficacy analysis using the mITT population and safety analysis for the safety population.  2. Sample size calculation  The study is an exploratory study with 20 planned to observe 20 cases.  3. Statistical analysis method  The specific statistical analysis methods will be detailed in an independent statistical analysis plan, briefly described in the protocol below:  3.1 General statistical considerations  For continuous data, mean, standard deviation, interquartile spacing, maximum and minimum value are described, and classified data are described by frequency and composition ratio.  3.2 Primary efficacy endpoint  Patients with a PEPI score of 0, as a percentage of the total number of patients analyzed, and their 95% CI;  3.3 Secondary efficacy endpoints  The percentage of patients with complete cell cycle arrest (CCCA: Ki-672.7%) and their 95% CI;  Pathological complete response rate (pCR: ypT 0 / is ypN0): percentage of patients in the total analyzed set and 95% CI;  Objective response rate (ORR): the percentage of complete response (CR) and partial response (PR) in the total number of analyzed patients and its 95% CI;  Disease control rate (DCR): patients with complete response (CR), partial remission (PR) and stable disease (SD), a percentage of the total number analyzed, and 95% CI;  EFS: Time from randomization (or enrollment) to the first occurrence (whichever occurs first): failure to undergo potentially curative surgery, local or distant invasive recurrence after radical mastectomy, new breast cancer or secondary malignancy, death from any cause;  Rate of breast-conserving surgery: the proportion of patients who were judged unsuitable by the investigator before randomization (or enrollment) meeting the criteria for breast-conserving surgery after completion of neoadjuvant therapy;  3.4 Safety endpoint  Descriptive statistics were made for the occurrence of adverse events and laboratory tests, electrocardiogram, vital signs, and physical examinations. Adverse events will be classified by the National Cancer Institute (NCI) version 4.03  Level 1 (CTC-AE) is graded and coded according to the MedDRA dictionary. The adverse events will be summarized based on the system organ classification and preferred terms. |
| stages of research  stages of research | This study is divided into: screening period, treatment period, end of treatment and no event, survival follow-up.  1. Screening period  After obtaining informed consent, eligible patients were screened for the trial by medical history, physical examination, laboratory examination, and tumor evaluation.  2. Treatment period  Patients eligible for screening received sitabenamine combined with exemestane (premenopausal + OFS) for sitabenamine tablets: 30mg twice a week (d1, d4, d8, d11, d15, d18). Take 30mg (6 tablets) orally for 30 minutes after the meal.  25mg each time, once a day, orally after meals.  OFS drugs: used according to clinical selection  The above treatment is a treatment cycle every 3 weeks for 4-6 months of drug treatment, during which disease progression (PD), other cancer drug treatment, surgery or radiotherapy for primary or metastatic disease, pregnancy, or other medical decision of the study is stopped, and the subsequent treatment is determined by the investigator.  If intolerable toxicity occurs during treatment, follow the dose adjustment protocol. After the completion of neoadjuvant therapy, the investigator conducted the evaluation. For operable patients, surgical treatment was given 4-6 weeks after the last dose of neoadjuvant therapy, and those who were willing and eligible underwent breast-conserving surgery.postoperative radiotherapy and the radiotherapy regimen are determined by the investigator. No postoperative adjuvant treatment plan, surgery  Post-systemic therapy is allowed.  After completion of neoadjuvant therapy, the investigator assessed as inoperable, and the subsequent treatment plan is agreed by the investigator and the patient.  3. End of treatment visit  Efficacy evaluation time: within ± 7 days after the last treatment for subjects who ended treatment due to disease progression or completed neoadjuvant treatment; within 7 days after the departure date for subjects who withdrew early because of the other reasons.  4. Event-free survival follow-up  1) From the end of treatment visit, until disease relapse or death (whichever occurs first). Telephone follow-up is allowed. Disease progression / relapse and treatment regimen were recorded.  2) Follow-up every 3 months (± 7d). Follow-up visits occurred every 6 months (± 7d) from 2 years after completion of treatment. |
